# Biosynthesis of the oxygenated diterpene nezukol in the medicinal plant *Isodon rubescens* is catalyzed by a pair of diterpene synthases

**DOI:** 10.1371/journal.pone.0176507

**Published:** 2017-04-26

**Authors:** Kyle A. Pelot, Lynne M. Hagelthorn, J. Bennett Addison, Philipp Zerbe

**Affiliations:** 1 Department of Plant Biology, University of California-Davis, Davis, California, United States of America; 2 Department of Chemistry, University of California-Davis, Davis, California, United States of America; NARO Institute of Agrobiologial Sciences, JAPAN

## Abstract

Plants produce an immense diversity of natural products (i.e. secondary or specialized metabolites) that offer a rich source of known and potentially new pharmaceuticals and other desirable bioproducts. The Traditional Chinese Medicinal plant *Isodon rubescens* (Lamiaceae) contains an array of bioactive labdane-related diterpenoid natural products. Of these, the *ent*-kauranoid oridonin is the most prominent specialized metabolite that has been extensively studied for its potent antimicrobial and anticancer efficacy. Mining of a previously established transcriptome of *I*. *rubescens* leaf tissue identified seven diterpene synthase (diTPSs) candidates. Here we report the functional characterization of four *I*. *rubescens* diTPSs. IrTPS5 and IrTPS3 were identified as an *ent*-copalyl diphosphate (CPP) synthase and a (+)-CPP synthase, respectively. Distinct transcript abundance of IrTPS5 and the predicted *ent*-CPP synthase IrTPS1 suggested a role of IrTPS5 in specialized *ent*-kaurene metabolism possibly en route to oridonin. *Nicotiana benthamiana* co-expression assays demonstrated that IrTPS4 functions sequentially with IrTPS3 to form miltiradiene. In addition, IrTPS2 converted the IrTPS3 product (+)-CPP into the hydroxylated tricyclic diterpene nezukol not previously identified in *I*. *rubescens*. Metabolite profiling verified the presence of nezukol in *I*. *rubescens* leaf tissue. The proposed IrTPS2-catalyzed reaction mechanism proceeds via the common ionization of the diphosphate group of (+)-CPP, followed by formation of an intermediary pimar-15-en-8-yl^+^ carbocation and neutralization of the carbocation by water capture at C-8 to yield nezukol, as confirmed by nuclear magnetic resonance (NMR) analysis. Oxygenation activity is rare for the family of class I diTPSs and offers new catalysts for developing metabolic engineering platforms to produce a broader spectrum of bioactive diterpenoid natural products.

## Introduction

Plant natural products, also called specialized or secondary metabolites, are a valuable but underutilized source for drug discovery [1, 2]. Diterpenoids form a diverse group of metabolites with essential functions in plant development and ecological adaptation [3], and some diterpenoids are of economic importance as bioproducts. This includes approved therapeutics, such as the chemotherapeutic drug taxol from the pacific yew tree (*Taxus brevifolia*) [4], the cAMP-regulating agent forskolin from *Coleus forskohlii* [5], and ingenol mebutate from *Euphorbia peplus* for the treatment of actinic keratosis [6]. Advanced analytical and engineering technologies pave the way to developing enzymatic biomanufacturing systems that can help to overcome the often limited availability of plant diterpenoids by chemical synthesis or extraction from predominantly not cultivable medicinal plants [7]. As a key to enabling these approaches, the discovery of novel bioactive diterpenoids and the corresponding biosynthetic genes and enzymes has attracted increasing attention in recent years, providing the prerequisite resources for devising enzyme-based production platforms [8].

*Isodon rubescens* (Lamiaceae; known as ‘dōng líng cǎo’ in Mandarin) is a medicinal plant native to Eastern China. Leaves of *I*. *rubescens* have traditionally been used to treat respiratory and gastrointestinal bacterial infections, inflammation and malignant tumors [9]. The more than 100 species of the genus *Isodon* produce a large diversity of over 500 different diterpenoids, primarily comprised of labdane-related products including *ent*-kaurenes, abietanes, pimaranes and clerodanes [9]. The major bioactive constituent of *I*. *rubescens* is the *ent*-kauranoid diterpenoid oridonin. Oridonin and derivatives thereof have been demonstrated to exert a broad spectrum of antitumor activities, making it an attractive lead compound for cancer therapies [10–12]. Despite its potential biopharmaceutical application, diterpenoid metabolism in species of *Isodon* has remained largely unresolved, with the exception of two diterpene synthases (diTPSs) from *I*. *eriocalyx* that produce copalyl diphosphate (CPP) [13].

The family of diTPSs facilitates the key committed reactions in diterpenoid metabolism by converting the central precursor geranylgeranyl diphosphate (GGPP) into an array of distinct typically polycyclic scaffolds [8, 14]. Downstream stereo- and regio-selective functional modification of these scaffolds predominantly catalyzed by cytochrome P450-dependent monooxygenases (P450s) then gives rise to more than 10,000 natural products, the majority of which represent labdane-related diterpenoids [14, 15]. In angiosperms, labdane-related diterpenoids are formed in modular metabolic networks, with pairs of monofunctional class II and class I diTPSs at their core [8, 16]. First, class II diTPSs catalyze the protonation-initiated cyclization of GGPP into bicyclic prenyl diphosphates of *ent*, normal (+) or *syn* stereochemistry [14]. Second, class I diTPSs cleave the diphosphate moiety and facilitate a variety of cyclization and rearrangement reactions to convert the resulting carbocation. While, neutralization of the carbocation is most commonly achieved by terminal deprotonation, diTPSs capable of stabilizing the intermediary carbocation by regio-specific water capture have also been demonstrated in various species, spanning mosses, gymnosperms and angiosperms [8]. The majority of these enzymes represent class II diTPSs which convert GGPP into hydroxylated prenyl diphosphate intermediates with the oxygen function predominantly at carbon eight (C-8) [17–24]. By contrast, fewer examples of position-specific hydroxylation reactions during class I diTPS catalysis have been described in bifunctional class I/II diTPSs and monofunctional class I diTPSs [19, 24–28]. Structure-guided protein mutagenesis studies further illustrated that the capacity of diTPSs for carbocation neutralization by water capture is governed by as little as a single active site residue, highlighting the ease with which such functions may have evolved across the plant kingdom [29–33].

Building on an earlier genomics-enabled gene discovery study that identified seven diTPS candidates [20], we here describe the cloning and functional characterization of four diTPSs (IrTPS2-5) that produce *ent*-CPP as the predicted precursor of bioactive kauranoid diterpenoids, as well as nezukol (8β-hydroxy-isopimar-15-ene), a hydroxylated diterpene not previously known to occur in *I*. *rubescens*.

## Results

### Identification of diterpene synthases

**Fig 1:**
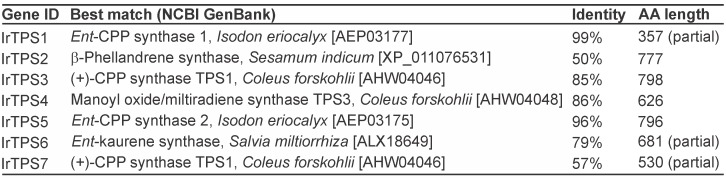
Diterpene synthases identified in the leaf transcriptome of *Isodon rubescens*.

To gain deeper insight into the biosynthesis of bioactive diterpenoids in *I*. *rubescens*, we previously developed leaf transcriptome assemblies [20]. Mining this resource revealed seven candidate diTPSs with best matches to known labdane-related diterpene synthases (Fig 1). Furthermore, two short transcript fragments (<800 bp) were identified that may represent additional diTPS genes, but lacked any significant matches in the NCBI GenBank database. Of the identified diTPSs transcripts, four (IrTPS1, 3, 5 and 7) were predicted as class II diTPSs based on the presence of the characteristic DxDD signature motif. Conversely, IrTPS2, 4 and 6 featured the conserved DDxxD and NDX_2_(S/T)X_3_E motifs relevant for catalysis in class I diTPSs. IrTPS1 and IrTPS5 displayed sequence similarity of higher than 95% to previously reported putative *ent*-CPP synthases from *I*. *eriocalyx* [13], while IrTPS3 was most similar to a (+)-CPP synthase from *Coleus forskohlii* [22]. The class I diTPSs IrTPS4 and IrTPS6 shared high sequence similarity with a manoyl oxide/miltiradiene synthase from *C*. *forskohlii* [22] and a *Salvia miltiorrhiza ent*-kaurene synthase, respectively [23]. Although with low identity scores, IrTPS7 matched most closely with the sequence of *C*. *forskohlii* (+)-CPP synthase, while IrTPS2 showed no significant protein sequence identity to known diTPSs (<50%) with the monoterpene synthase β-phellandrene synthase of *Sesamum indicum* as best match.

**Fig 2:**
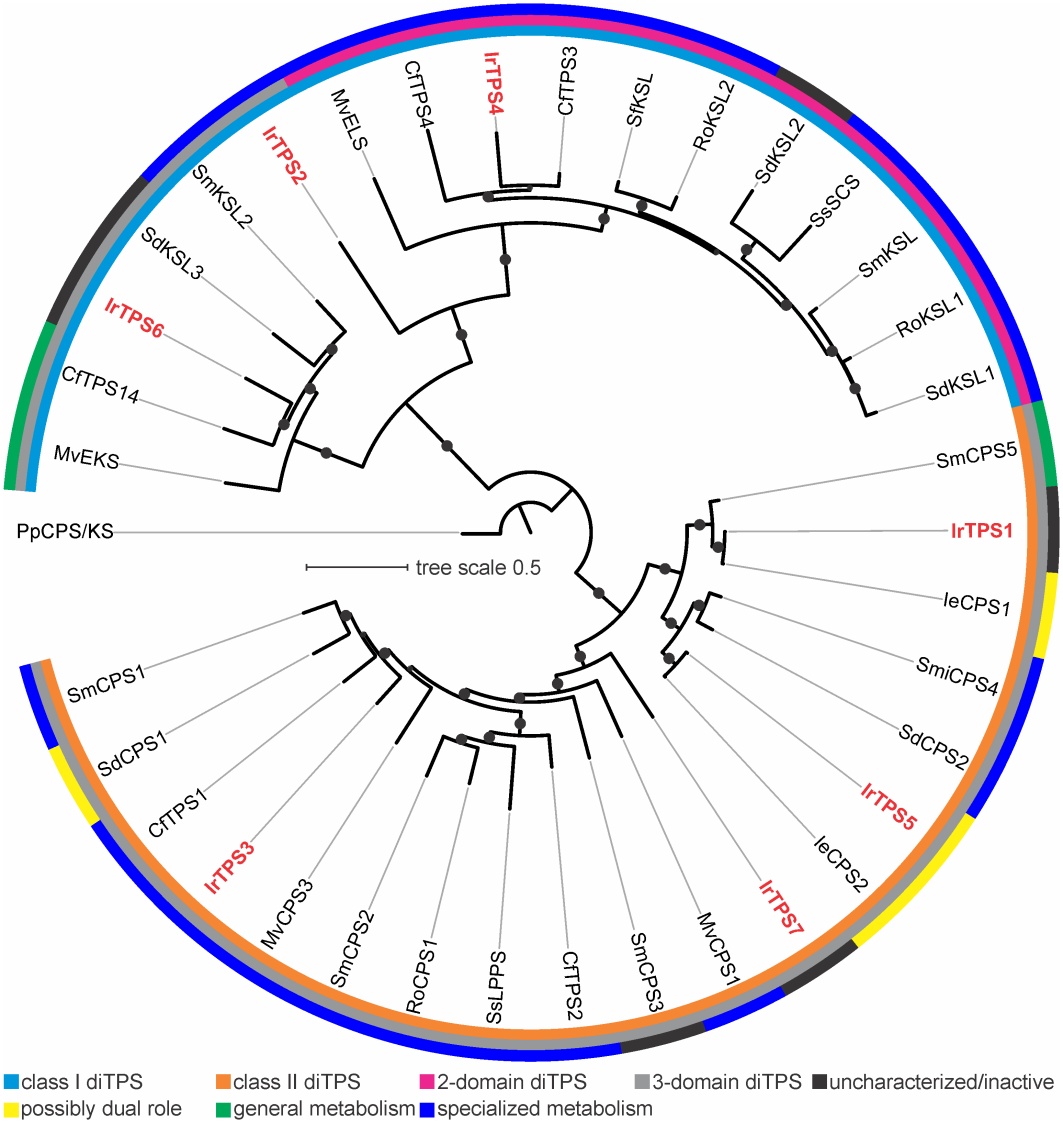
Phylogeny of Lamiaceae diterpene synthases. The illustrated maximum-likelihood tree was constructed with known diTPSs from species of the Lamiaceae family, using the ancestral bifunctional class I/II diTPS *Physcomitrella patens ent*-kaurene/kaurenol synthase (PpCPS/KS) as an outgroup. Different domain architecture and association with general or specialized metabolism (or both) are highlighted. Bootstrap (500 repetitions) confidence values over 80% are illustrated at branch points. Abbreviations and GenBank accession numbers are listed in S1 Table.

A phylogeny with known diTPSs from the Lamiaceae family (Fig 2) placed IrTPS1 and IrTPS5 closely with the putative *ent*-CPP synthases of IeCPS1 and IeCPS2 of *I*. *eriocalyx*, supporting a close functional relatedness. Consistent with our functional prediction, IrTPS3 clustered most closely with a group of class II diTPSs that predominantly produce (+)-CPP [21–23]. Among the class I diTPSs, IrTPS6 was placed within a clade that includes primarily *ent*-kaurene synthases *bona fide* involved in gibberellin biosynthesis of general metabolism. IrTPS4 belonged to a clade of class I diTPSs with an unusual 2-domain structure, all known members of which show functions in specialized metabolism [21–23]. Notably, our sequence phylogeny placed IrTPS2 on a separate branch adjacent to the clade of specialized class I diTPSs (Fig 2). Together with the low protein sequence similarity of less than 50% to known plant diTPSs, this distant clade association suggested a substantial evolutionary divergence and probably distinct activity of IrTPS2, making it a high priority candidate for biochemical characterization.

### Functional characterization of diterpene synthases

**Fig 3:**
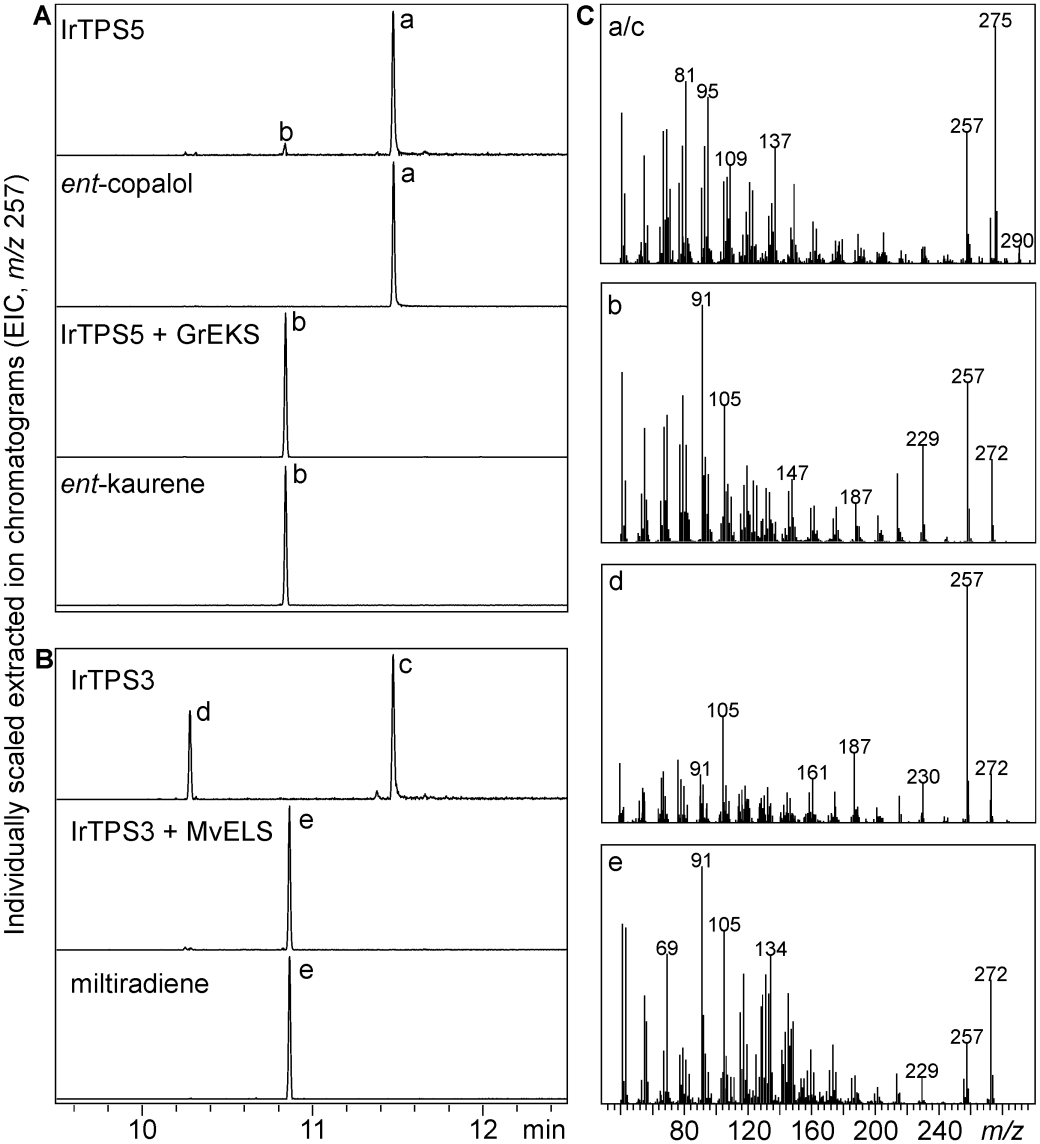
Biochemical characterization of IrTPS5 and IrTPS3. GC-MS analysis of extracted enzyme products resulting from *Agrobacterium*-mediated transient *N*. *benthamiana* co-expression assays of the class II diTPSs IrTPS5 (**A**) and IrTPS3 (**B**) with the class I diTPSs *Grindelia robusta ent*-kaurene synthase (GrEKS) or *Marrubium vulgare* epoxy-labdane/miltiradiene synthase (MvELS). Reaction products are depicted as extracted ion chromatograms (EIC, *m*/*z* 257) with corresponding mass spectra (**C**) for products: a, *ent*-copalol (i.e. dephosphorylated CPP); b, *ent*-kaurene; c, (+)-copalol; d, unidentified diterpene; e, miltiradiene.

Of the seven diTPSs identified in the *I*. *rubescens* leaf transcriptome, only the class II diTPSs IrTPS5 and IrTPS3 and the class I diTPSs IrTPS2 and IrTPS4 could be retrieved as full-length (FL) cDNA for further functional analysis. To determine the enzymatic activity of these diTPSs, we conducted *in vivo* combinatorial assays using transient *Agrobacterium*-mediated co-expression in *Nicotiana benthamiana* [20]. Due to the natural pairwise activity of monofunctional class II and class I diTPSs in angiosperm labdane biosynthesis [16, 20, 34, 35], combinatorial expression of class II and class I diTPSs can be used to determine the identity and stereochemistry of enzyme products [21, 23, 24, 36–45]. To this end, we co-expressed IrTPS5 and IrTPS3 with previously characterized class I diTPSs that exhibited substrate-specificity to *ent*-CPP (*Grindelia robusta ent*-kaurene synthase, GrEKS [37]) and (+)-CPP (*Marrubium vulgare* epoxy-labdane/miltiradiene synthase, MvELS [21]), respectively. Transient expression of IrTPS5 alone yielded copalol (product a) as verified by comparison to an enzyme-produced standard (Fig 3A). Production of copalol representing the dephosphorylated form of CPP is commonly observed in transient *N*. *benthamiana* co-expression assays of class II diTPSs and is presumably caused by endogenous phosphatases that dephosphorylate the class II diTPS products [17, 18, 21, 36, 37]. Subsequent co-expression of IrTPS5 with GrEKS afforded *ent*-kaurene (product b) as the sole product, while no product formation was observed after co-expression with MvELS. Minor amounts of *ent*-kaurene were also detected when expressing IrTPS5 alone, and presumably result from the activity of the endogenous *N*. *benthamiana ent*-kaurene synthase.

Transient expression of IrTPS3 also resulted in the formation of copalol (product c) and a second unidentified diterpene (product d). This secondary product is likely resulting from thermal degradation of copalol during GC-MS analysis as previously observed in transient expression analyses of other class II diTPSs [21, 24, 37, 46]. In contrast to IrTPS5, sequential activity of IrTPS3 was only detected with MvELS yielding miltiradiene (product e), whereas no product formation was detected after co-expression with GrEKS (Fig 3B). Together, these results identified IrTPS5 and IrTPS3 as an *ent*-CPP synthase and (+)-CPP synthase, respectively.

**Fig 4:**
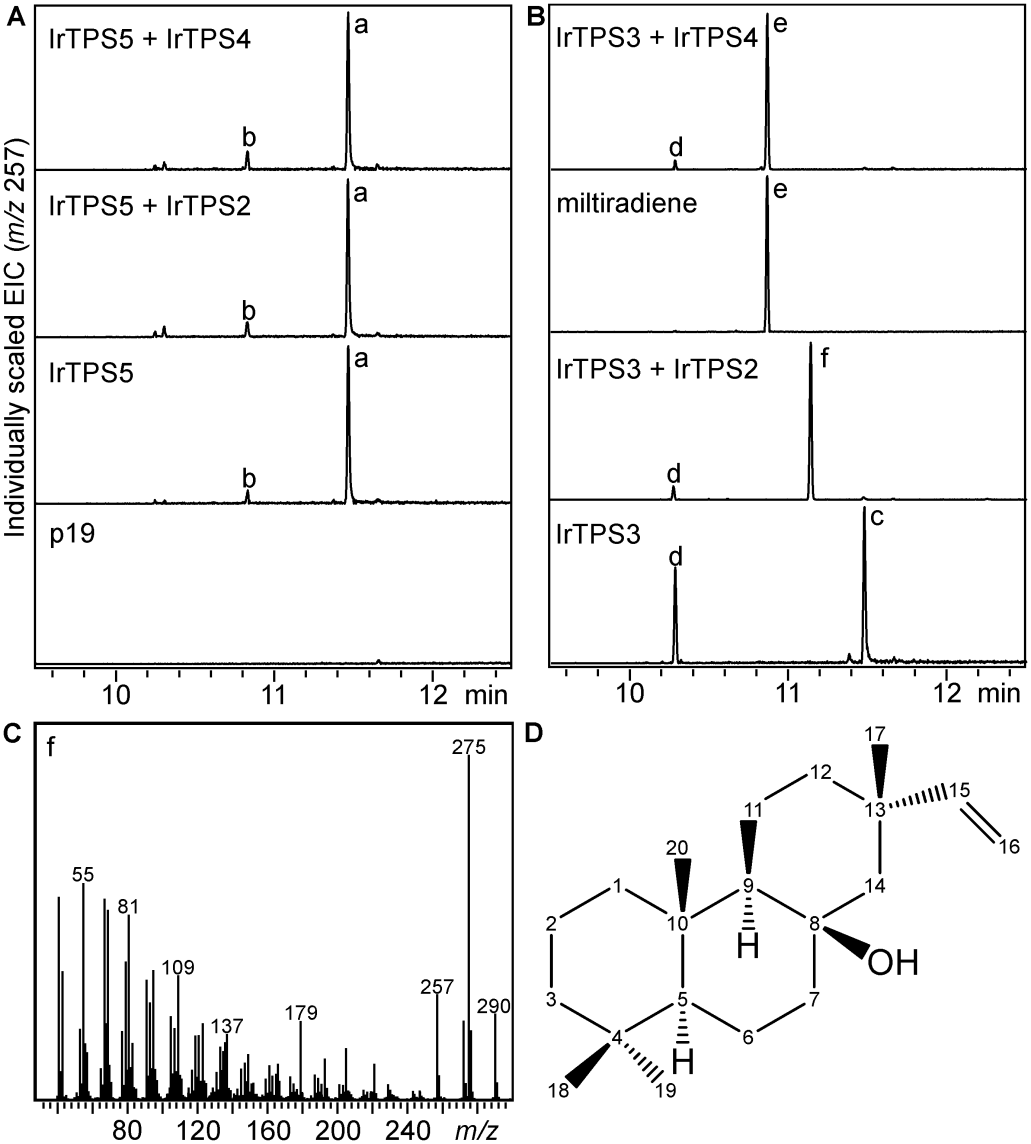
Biochemical characterization of IrTPS4 and IrTPS2. Illustrated are extracted ion chromatograms (EIC, *m*/*z* 257) of products obtained in *Agrobacterium*-mediated transient *Nicotiana benthamiana* co-expression assays of IrTPS2 and IrTPS4 with IrTPS5 (**A**) and IrTPS3 (**B**), respectively. Expression of IrTPS3, IrTPS5 and the RNA silencing suppressor protein p19 served as controls. Reaction products: a, *ent*-copalol (i.e. dephosphorylated CPP); b, *ent*-kaurene; c, (+)-copalol; d, unidentified diterpene; e, miltiradiene; f, nezukol. (**C**) Mass spectrum of the product resulting from coupled activity of IrTPS3 and IrTPS2 with significant similarity to reference mass spectra of 8β-hydroxy-isopimar-15-ene or nezukol (product f). (**D**) Structure of nezukol as verified by 1D and 2D nuclear magnetic resonance (NMR) analysis.

Having identified the catalytic activities of IrTPS5 and IrTPS3, we next probed the function of the class I diTPSs by co-expressing IrTPS3 or IrTPS5 with IrTPS4 and IrTPS2. No activity of IrTPS2 or IrTPS4 was found when co-expressed with the *ent*-CPP synthase IrTPS5 (Fig 4A). Sequential reaction of IrTPS4 and the (+)-CPP synthase IrTPS3 afforded miltiradiene (product e) as a single product when compared to the product profile of IrTPS3 alone and an authentic miltiradiene standard (Fig 4B). Co-expression of IrTPS2 and IrTPS3 lead to a distinct product (product f) which displayed a fragmentation pattern characteristic of the diterpene 8β-hydroxy-isopimar-15-ene (also coined nezukol) with dominant mass ions of *m*/*z* 290, *m*/*z* 275, *m*/*z* 257, *m*/*z* 179, *m*/*z* 137 and *m*/*z* 109 (Fig 4C) [47]. The minor unidentified diterpene (product d) detected in the individual IrTPS3 expression, was also present at low quantities in combinations of IrTPS3 with IrTPS4 and IrTPS2.

To unambiguously confirm the structure of the IrTPS2 product via nuclear magnetic resonance (NMR) analysis, a suitable amount was produced using an engineered *E*. *coli* system in which IrTPS2 was co-expressed with a (+)-CPP synthase (AgAS:D621A) and a GGPP synthase from *Abies grandis* [48]. Following hexane extraction and purification by silica chromatography and HPLC, 1D and 2D NMR spectra were acquired, and verified the IrTPS2 product as nezukol (Fig 4D; S1 Fig) consistent with previous spectral references [47, 49, 50]. Normal (+) stereochemistry of nezukol at positions C-17, C-18 and C-20 can be inferred from the substrate (+)-CPP and was also supported by selective 1D nuclear Overhauser effect (nOe) difference analyses that showed interactions between the methyl groups at C-17 and C-20, as well as C-18 and C-20 (S2 Fig). In addition, quantum chemical calculations supported a β-configuration of the hydroxyl group at C-8.

### Abundance of nezukol *in planta*

**Fig 5:**
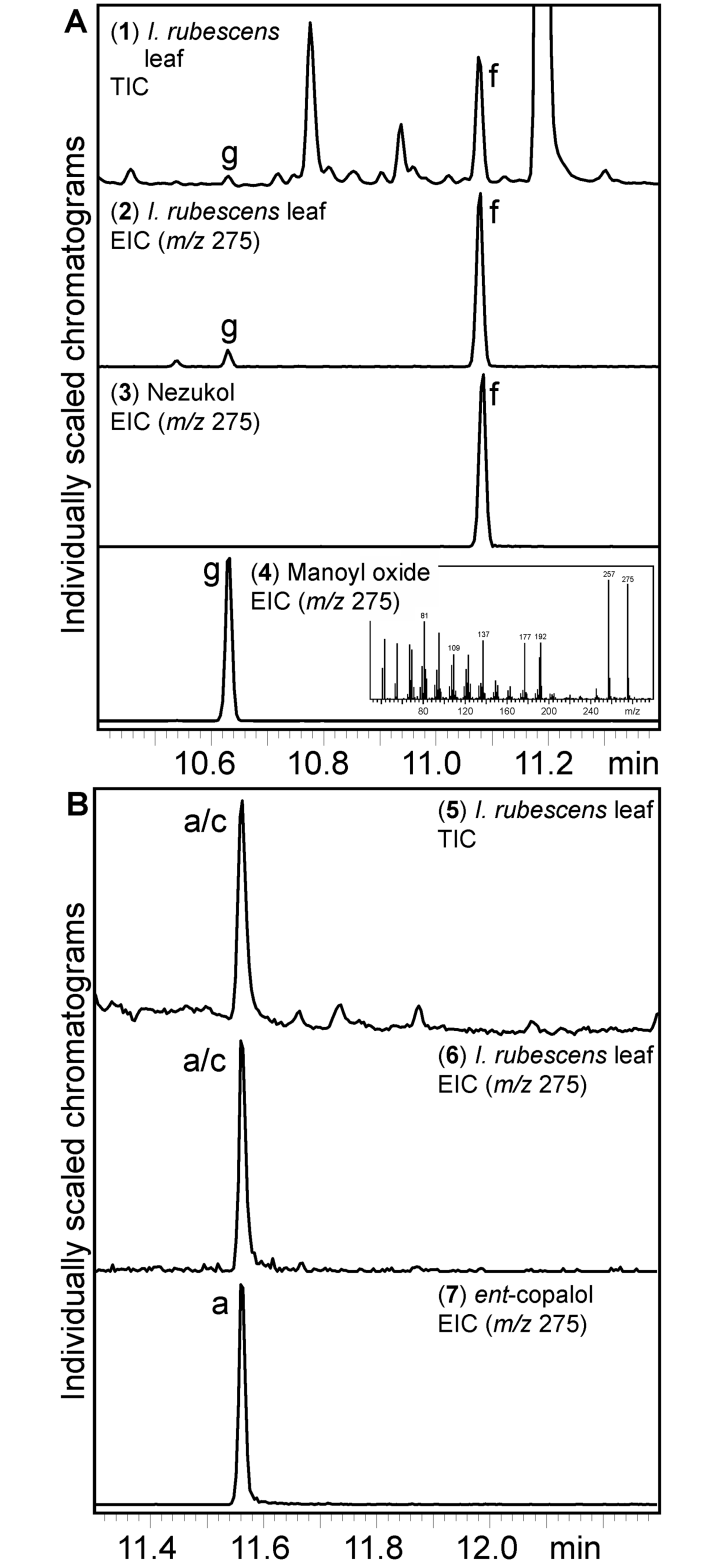
Nezukol abundance in *Isodon rubescens* leaf tissue. GC-MS analysis of ethyl acetate/hexane fractionated hexane extracts of *I*. *rubescens* leaf tissue. (**A**) Total ion chromatogram (TIC, **1**) and extracted ion chromatogram (EIC, *m*/*z* 275, **2**) of a 20:80 (v/v) ethyl acetate/hexane fraction containing the compounds nezukol (product f) and manoyl oxide (product g). Compounds were identified by comparison to purified enzyme products (**3–4**). (**B**) TIC (**5**) and EIC (*m*/*z* 275, **6**) of a 40:60 (v/v) ethyl acetate/hexane fraction containing copalol (product a or c) as compared to enzyme-produced *ent*-copalol (**7**).

Nezukol had not previously been reported to occur in species of the genus *Isodon*. To investigate its presence in leaves of *I*. *rubescens*, we prepared hexane extracts of *I*. *rubescens* leaf tissue, which were further fractionated over silica gel using an ethyl acetate/hexane gradient and analyzed by GC-MS (Fig 5). Albeit at low abundance, nezukol (product f) was detected in the 20:80 (v/v) ethyl acetate/hexane fraction of leaf extracts as verified by comparison to the purified IrTPS2 product. In addition, trace amounts of manoyl oxide (product g) were detected in this fraction. In all known examples, biosynthesis of manoyl oxide requires the initial formation of labda-13-en-8-ol diphosphate (LPP) by a class II diTPS. In *I*. *rubescens* this function could be served by IrTPS7 which phylogenetically clustered with known LPP synthases (Fig 2). However, the FL sequence of IrTPS7 could not be retrieved preventing a further testing of this hypothesis within the scope of this study. Furthermore, copalol (product a or c) was present in the 40:60 (v/v) ethyl acetate/hexane fraction as identified by the IrTPS5 product *ent*-copalol (i.e. dephosphorylated CPP). However, the absolute stereochemistry of copalol *in planta* could not be determined.

### Gene expression analysis of *I*. *rubescens* diterpene synthases

**Fig 6:**
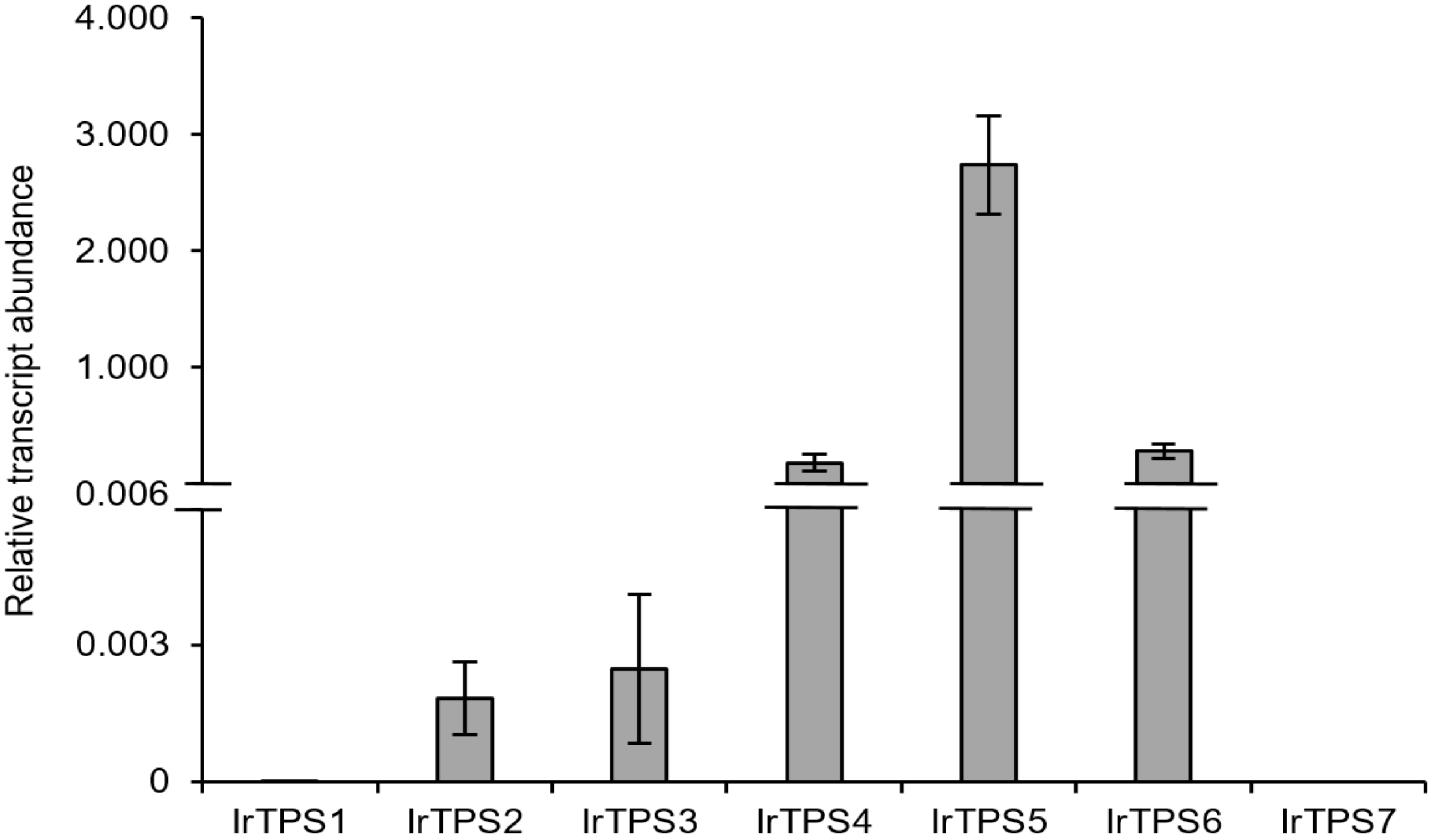
Relative transcript abundance of *I*. *rubescens* diTPSs in leaf tissue. Relative transcript abundance was measured by quantitative real-time PCR (qRT-PCR) and normalized to actin as internal reference gene. Error bars represent standard errors based on duplicate measurements of three biological replicates. Reaction specificity was confirmed by melt curve analysis of each sample and sequence verification of representative amplicons.

Transcript abundance of all seven identified diTPSs in *I*. *rubescens* leaf tissue was assessed by quantitative real-time PCR (qRT-PCR) analysis (Fig 6). *IrTPS5* encoding an *ent*-CPP synthase showed the highest transcript abundance, whereas the predicted *ent*-CPP synthase *IrTPS1* and the putative CPP or LPP synthase *IrTPS7* were detected at only trace levels. Low transcript abundance was also observed for the nezukol synthase *IrTPS2* and the (+)-CPP synthase *IrTPS3*, consistent with the low abundance of nezukol *in planta* (Fig 5). Transcripts of the miltiradiene synthase *IrTPS4* and the putative *ent*-kaurene synthase *IrTPS6* were again higher, but approximately 16-fold and 10-fold lower, respectively, as compared to *IrTPS5*.

## Discussion

The diversity and bioactivity of plant diterpenoids provides a vast chemical space that can be explored for the discovery of biopharmaceutical lead compounds to aid the urgently needed development of novel and improved drugs [2, 8]. Impractical isolation of diterpenoids from natural sources or uneconomic chemical synthesis are limiting the availability of diterpenoid natural products, and only a few diterpenoid-based pharmaceuticals, including taxol, forskolin and ingenol mebutate are currently manufactured at commercial scale [8]. With rapid advances in elucidating diterpenoid-biosynthetic enzymes in an ever-increasing number of plant species, pathway engineering platforms can provide alternative means to make available the broad spectrum of diterpenoids produced in the many often rare or endangered non-model medicinal plants that produce bioactive diterpenoids.

**Fig 7:**
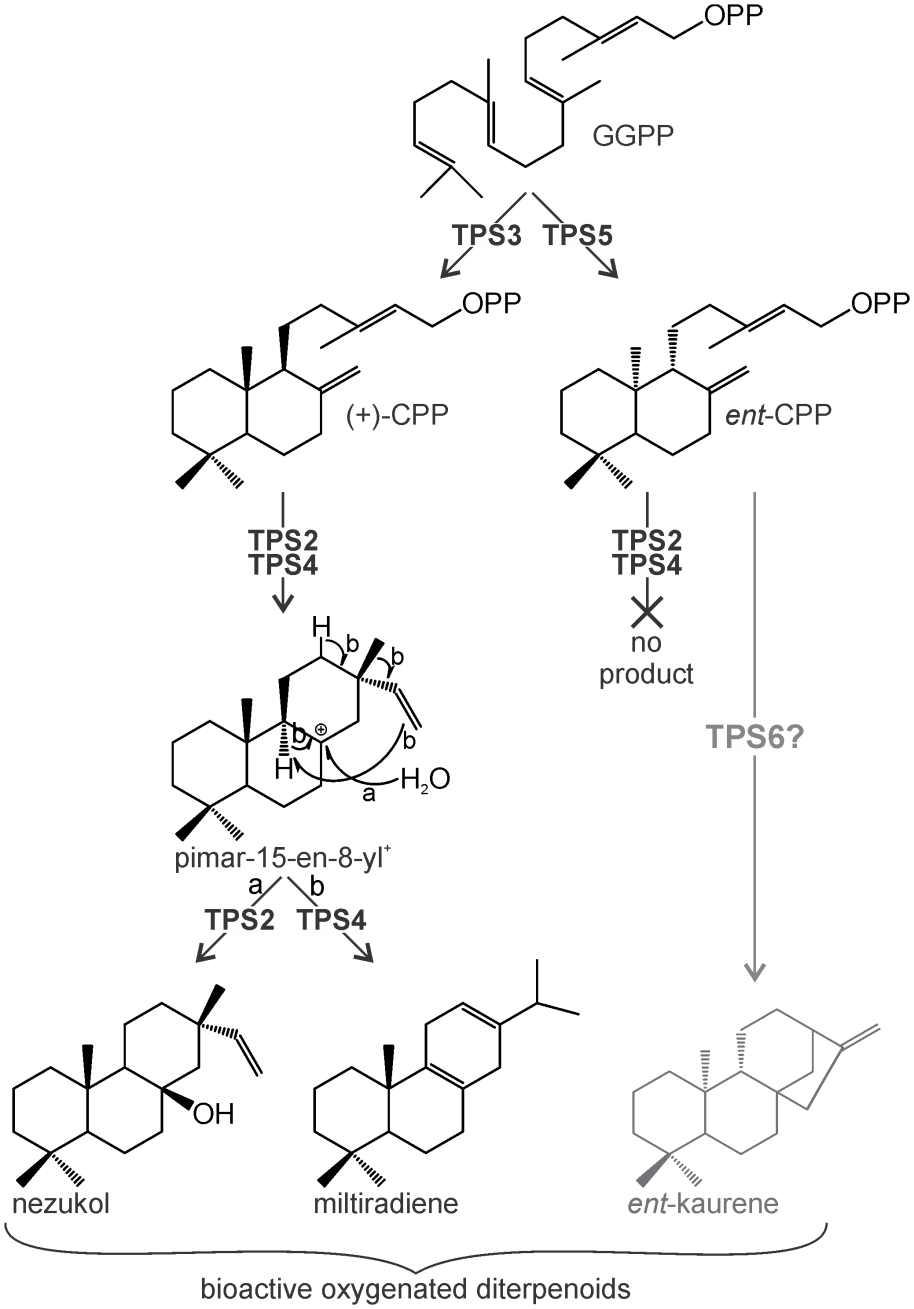
Proposed reaction pathways en route to nezukol and other *I*. *rubescens* diterpenes. Following the common framework of labdane biosynthesis in angiosperms, pairs of monofunctional class II and class I diTPSs were identified that produce distinct diterpene scaffolds. First, IrTPS3 and IrTPS5 transform the central precursor geranylgeranyl diphosphate (GGPP) into the bicyclic prenyl diphosphate intermediates (+)-copalyl diphosphate (CPP) and *ent*-CPP, respectively. Second, the class I diTPSs IrTPS2 and IrTPS4 catalyze diphosphate ionization to form a common pimar-15-en-8-yl^+^ carbocation. IrTPS4-catalyzed rearrangement of this carbocation by 1,6 proton transfer and 1,2 methyl migration followed by deprotonation at C-13 yields miltiradiene. In contrast, IrTPS2 facilitates the direct neutralization of the carbocation by water capture at C-8 to form the hydroxylated diterpene nezukol.

The diversity of bioactive diterpenoids in *I*. *rubescens* has made this Traditional Chinese Medicinal plant an attractive target for drug discovery, spearheaded by the potential anticancer agent oridonin [9]. Using *in vivo* combinatorial expression analysis, metabolite profiling and gene expression studies, functional characterization of four *I*. *rubescens* diTPSs identified catalytic activities presumably en route to oridonin or related kauranoids and a previously hidden *I*. *rubescens* metabolite nezukol (Fig 7). Thus, this study provides an example of the power of genomics-enabled gene and pathway elucidation for the discovery of novel plant natural products in non-model plants.

The kauranoid oridonin has been shown to be the major constituent of *I*. *rubescens* foliar tissue [9]. Oridonin biosynthesis predictably recruits the consecutive activity of a class II and a class I diTPS that form the *ent*-CPP and *ent*-kaurene core scaffolds, thus sharing these key intermediates with the formation of gibberellin phytohormones (Fig 7). High transcript abundance of IrTPS5 (Fig 6) correlates with the previously demonstrated abundance of oridonin in *I*. *rubescens* [9] and together with the *ent*-CPP synthase activity of IrTPS5 (Fig 3) may support a role in the biosynthesis of oridonin or related kauranoids. Alternatively, IrTPS5 may have a dual function in both gibberellin and oridonin biosynthesis in a similar fashion as demonstrated for the *ent*-CPP synthase (SrCPS) and *ent*-kaurene synthase (SrKS) of *Stevia rebaudiana* involved in gibberellin metabolism and biosynthesis of the specialized diterpenoid steviol [51]. In addition, an *ent*-CPP synthase and an *ent*-kaurene synthase function may be proposed for IrTPS1 and IrTPS6, respectively, as based on blast matches and phylogenetic relatedness with known diTPSs. However, low transcript abundance and lack of FL sequences prevented biochemical characterization of the encoded enzymes.

Consistent with our phylogenetic predictions (Fig 2), transient *N*. *benthamiana* combinatorial assays further demonstrated the formation of the specialized metabolite miltiradiene through the sequential function of the (+)-CPP synthase IrTPS3 and the miltiradiene synthase IrTPS4 (Figs 3 and 4). As described for other miltiradiene synthases, IrTPS4 will facilitate the cleavage of the (+)-CPP diphosphate group to form the central pimar-15-en-8-yl^+^ carbocation, which will undergo 1,6 proton transfer, 1,2 methyl migration and terminal deprotonation to afford miltiradiene [52]. Miltiradiene is broadly distributed among members of the Lamiaceae and serves as a key intermediate in the biosynthesis of, for example, bioactive tanshinones in *S*. *miltiorrhiza* [23, 53] and the food preservative carnosic acid in rosemary (*Rosmarinus officinalis*) [46, 54, 55].

Indeed, several oxygenated abietane-type diterpenoids, such as the rubesanolides, have been identified in *I*. *rubescens* that may derive from miltiradiene [9, 56, 57].

An expansive evolutionary diversification and possibly distinct enzymatic function of IrTPS2 was indicated by the low sequence similarity compared to known diTPSs and placement of the encoded enzyme on a distant class I diTPS branch in a phylogenetic tree of Lamiaceae diTPSs (Fig 2). This hypothesis was confirmed by co-expression of IrTPS3 and IrTPS2 that yielded the hydroxylated diterpene nezukol (8β-hydroxy-isopimar-15-ene) as verified by 1D and 2D NMR analysis and identification of nezukol in leaf tissue (Figs 4 and 5). Discovery of IrTPS2 adds a new catalyst to the diverse family of plant diTPSs that is capable of facilitating the biosynthesis of a hydroxylated diterpene structure. Numerous examples of oxygenated products have been demonstrated for both gymnosperm and angiosperm class II diTPSs in recent years, including C-8- or C-9-hydroxylated bicyclic prenyl diphoposphate products [17–23, 58]. By contrast, there have been fewer reports of hydroxylation reactions catalyzed by the class I active site of diTPSs [19, 24–28]. These enzymes show a diversity of products containing hydroxyl groups at various positions, including the bifunctional class I/II diTPSs 16α-hydroxy-*ent*-kaurane synthase of *Physcomitrella patens*, labda-7,13*E*-dien-15-ol synthase of *Selaginella moellendorffii* and 13-hydroxy-8(14)-abietene synthases in several gymnosperm species [25–27]. Also, monofunctional class I enzymes such as sclareol synthase from *Salvia sclarea* [19] and 16α-hydroxy-*ent*-kaurane synthases from *Tripterygium wilfordii* and *Populus trichocarpa* have been identified [24, 28].

Similar to IrTPS4, the IrTSP2-catalyzed reaction will proceed via the pimar-15-en-8-yl^+^ carbocation following cleavage of the (+)-CPP diphosphate group. However, carbocation neutralization by water quenching at C-8 without prior rearrangement of the hydrocarbon scaffold will lead to nezukol as a final product (Fig 7). Although nezukol has not previously been reported in species of the genus *Isodon*, pimarane-related diterpenoids carrying oxygen functions at C-8 that could derive from nezukol have been reported, including forrestins isolated from *I*. *forrestii* and pimarane alcohol or acetoxy derivatives from *I*. *parvifolia* [9]. However, the intermediacy of nezukol in the formation these compounds is unclear, due to variation in the stereochemistry of the labdane skeleton and configuration of the hydroxyl group among these reported compounds. Beyond *I*. *rubescens*, nezukol has thus far predominantly been identified in needles and heartwood of various coniferous trees, including species of the genera *Podocarpus*, *Cryptomeria*, and *Cupressus* [47, 49, 59, 60], suggesting that this diTPS function evolved independently in several species; a prevalent feature of plant specialized metabolism. Notably, insight into the underlying mechanism has recently been achieved by site-directed mutagenesis of *Arabidopsis thaliana ent*-kaurene synthase by altering product specificity from *ent*-kaurene to 8α-hydroxy-*ent*-pimar-15-ene, thus highlighting how nezukol synthase activity may have evolved from diTPS progenitors of general metabolism [61, 62].

While the intermediary function of nezukol in *I*. *rubescens* diterpenoid metabolism requires further clarification, the discovery of IrTPS2 expands our knowledge of the broad catalytic space of the diTPS family that is not limited to the formation of hydrocarbon scaffolds, but extends to specific hydroxylation reactions that historically have been considered a prerogative of the vast family of P450 enzymes. This expanding catalytic range of diTPSs provides new targets for engineering of a broader spectrum of high-value diterpenoid bioproducts.

## Materials and methods

### Plant material

*Isodon rubescens* plants were purchased from the Horizon Herbs nursery (Williams, OR), and cultivated in a greenhouse under ambient photoperiod and ~22/17°C day/night temperature. *Nicotiana benthamiana* plants were cultivated from seeds in a Conviron PGR15 growth chamber with 16 h light at 80 μmol m^-2^ sec^-1^ irradiance and a 24/17°C day/night temperature cycle.

### Cloning of full-length cDNA

Transcriptome sequencing of *I*. *rubescens* leaf tissue and discovery of diTPS candidate genes have been described as part of an earlier study [19]. Total RNA from *I*. *rubescens* leaves was extracted using the Ambion RNAqueous-Micro kit (Thermo Fisher Scientific, Waltham, MA) and first-strand cDNA was reverse-transcribed using the SuperScript III First-Strand Synthesis System (Thermo Fisher Scientific, Waltham, MA) with oligo(dT)_20_ oligonucleotides. FL constructs of the targeted diTPS genes were amplified from cDNA using gene-specific oligonucleotides (S2 Table) and cloned into pJET (Thermo Fisher Scientific, Waltham, MA) for sequence verification. Subsequently, FL constructs were subcloned into the pLIFE33 expression vector for *Agrobacterium*-mediated transient expression in *N*. *benthamiana*. Additionally, a N-terminally truncated IrTPS2 construct lacking the predicted 45 amino acid plastidial transit peptide was amplified and transferred into the pET28b(+)vector (EMD Millipore, Billerica, MA) for expression in *E*. *coli*.

### Transient co-expression assays in *Nicotiana benthamiana*

For expression in *N*. *benthamiana*, FL constructs in the pLIFE33 vector were transformed into *A*. *tumefaciens* strain GV3101 by freeze-thaw. Resulting cells were grown at 28°C in LB media containing 50 mg L^-1^ of kanamycin, 30 mg L^-1^ of gentamicin and 10 mg L^-1^ of rifampicin. Cells were precipitated and resuspended to a final OD_600_ of 1.25 in 10 mM MES buffer with 10 mM MgCl_2_. Cultures were combined in equal volumes along with the silencing suppressor strain p19 [63]. After 1 h incubation of gentle shaking at 22°C, the abaxial side of the leaves of 6-week-old *N*. *benthamiana* plants were infiltrated. Five days post transfection, infected leaves were ground in liquid N_2_ and diterpene metabolites were extracted using hexane. GC-MS analysis of enzyme products was conducted on an Agilent 7890B GC interfaced with a 5977 Extractor XL MS Detector at 70 eV and 1.2 mL min^-1^ He flow, using a HP5-ms column (30 m, 250 μm i.d., 0.25 μm film) and the following GC parameters: 50°C for 2 min, 25°C min^-1^ to 300°C, hold 3 min with pulsed splitless injection at 250°C.

### Nuclear magnetic resonance (NMR) analysis

For NMR analysis of the IrTPS2 product, an amount in excess of 1 mg was produced by co-expression of the construct pET28b(+):IrTPS2 with the plasmids pGG*n*C, pGG and pIRS in *E*. *coli* BL21-C41 cells as previously described [48]. Inoculations were cultured at 37°C in 500 ml Terrific Broth medium to an OD_600_ of ~0.6 before protein expression was induced with 1 mM isopropyl-β-D-1-thiogalacto-pyranoside (IPTG) at 16°C for 72 h with the addition of 25 mM sodium pyruvate at 0, 24 and 48 h time points. The hexane extract was air dried and re-dissolved in 10 mL of hexane for purification on silica gel (70–230 mesh; Sigma-Aldrich, St. Louis, MO) using ethyl acetate/hexane as mobile phase at a stepwise gradient of 10%, 20%, 30%, 40% (5 mL each step). Nezukol-containing fractions were further HPLC purified using an Agilent 1100 HPLC system with a RRHD Eclipse Plus C18 column (2.1 x 50 mm) and an acetonitrile/dH_2_O mobile phase at a flow rate of 1 mL min^-1^. Purity of nezukol fractions was further verified by GC-MS analysis as described above. For NMR analysis, nezukol was dissolved in deuterated chloroform (CDCl_3_; Sigma-Aldrich, St. Louis, MO) containing 0.03% (v/v) tetramethylsilane (TMS). NMR 1D (^1^H, ^13^C, nOe) and 2D (HSQC, ^1^H-^1^H COSY, HMBC, H2BC) spectra were acquired on a Bruker Avance III 800 MHz spectrometer equipped with a 5 mm CPTCI cryoprobe. All NMR spectra were acquired with TopSpin 3.2 software (Bruker, Billerica, MA) and analyzed with MestReNova 11.0.2 software (Mestrelab Research, Santiago de Compostela, Spain). Chemical shifts (S1 Fig) were calibrated against known chloroform (^1^H 7.26 and ^13^C 77.0 ppm) signals, and compared to previously reported chemical shifts for nezukol [35,37,38].

### Leaf tissue metabolite analysis

Diterpene metabolites were extracted from flash frozen leaf tissue (~2–3 g) ground to a fine powder in liquid N_2_. Diterpene extraction was achieved with 50 mL of hexane under vigorous shaking over night at 16°C. The extract was air dried and resuspended in 2 mL of hexane, which was fractionated by silica gel (70–230 mesh; Sigma-Aldrich, St. Louis, MO) with an ethyl acetate/hexane mobile phase at a stepwise gradient of 10%, 20%, 30%, 40% (5 mL each step). The subsequent 1 mL fractions were concentrated to 200 μL for GC-MS analysis, as described above.

### Quantitative real-time PCR (qRT-PCR) analysis

Total leaf RNA was isolated as described above with the exception of additional DNase I treatment prior to cDNA synthesis. Quantitative real-time PCR reactions were performed on a Bio-Rad CFX96 Real-time system using iTaq SYBR Green Supermix (Bio-Rad, Hercules, CA) with gene specific oligonucleotides (S2 Table). Oligonucleotide specificity was verified by melt curve analysis and sequencing of representative amplicons. Relative transcript abundance was determined using efficiency corrected ΔCT values based on actin as the reference gene and duplicate measurements of three biological replicates.

### Phylogenetic analysis

Protein sequence alignments were generated using ClustalW and manually curated with Gblocks [50]. A maximum likelihood phylogenetic tree was generated using the PhyML server [51] with four rate substitution categories, LG substitution model, BIONJ starting tree and 500 bootstrap repetitions.

### Accession numbers

Nucleotide sequences of enzymes characterized in this study have been submitted to the GenBankTM/EBI Data Bank with accession numbers: IrTPS1 (KY661361), IrTPS2 (KX831650), IrTPS3 (KX831651), IrTPS4 (KX831652), IrTPS5 (KX831653), IrTPS6 (KY661362), IrTPS7 (KY661363).

## Supporting information

S1 Table Abbreviations and accession numbers of proteins used for phylogenetic analysis. (PDF)

S2 Table Oligonucleotides used in this study. (PDF)

S1 Fig NMR analysis of nezukol. (PDF)

S2 Fig Nezukol 1D selective nOe spectra of Me-20 and Me-17. (PDF)

## Abbreviations

CPP: copalyl diphosphate
diTPS: diterpene synthase
FL: full-length
GC-MS: gas chromatography-mass spectrometry
GGPP: geranylgeranyl diphosphate
HPLC: high-performance liquid chromatography
Ir: *Isodon rubescens*
