# Correction: Biosynthesis of the oxygenated diterpene nezukol in the medicinal plant *Isodon rubescens* is catalyzed by a pair of diterpene synthases

**DOI:** 10.1371/journal.pone.0224781

**Published:** 2019-10-30

**Authors:** Kyle A. Pelot, Lynne M. Hagelthorn, J. Bennett Addison, Philipp Zerbe

## Notice of republication

This article was republished on October 21, 2019, to correct the author list. Please download this article again to view the correct version.
