# Supplementary material for: Biosynthesis of the oxygenated diterpene nezukol in the medicinal plant Isodon rubescens is catalyzed by a pair of diterpene synthases
Source: PLoS One. 2017 Apr 26;12(4):e0176507. doi: 10.1371/journal.pone.0176507 (PMC5405970; doi:10.1371/journal.pone.0176507)
Supplement: S1 Table — (PDF) [file pone.0176507.s001.pdf]

**S1 Table. Abbreviations and accession numbers of proteins used for phylogenetic analysis**

| Protein | Function | Species | Accession No. |
|---|---|---|---|
| CfTPS1 | (+)-copalyl diphosphate synthase | *Coleus forskohlii* | KF444506 |
| CfTPS14 | *ent*-kaurene synthase | *Coleus forskohlii* | AGN70881 |
| CfTPS2 | labda-13-en-8-ol diphosphate synthase | *Coleus forskohlii* | KF444507 |
| CfTPS3 | miltiradiene / manoyl oxide synthase | *Coleus forskohlii* | KF444508 |
| CfTPS4 | miltiradiene / manoyl oxide synthase | *Coleus forskohlii* | KF444509 |
| IeCPS1 | *ent*-copalyl diphosphate synthase | *Isodon eriocalyx* | AEP03177 |
| IeCPS2 | *ent*-copalyl diphosphate synthase | *Isodon eriocalyx* | AEP03175 |
| IrTPS1 | uncharacterized diterpene synthase | *Isodon rubescens* | KY661361 |
| IrTPS2 | nezukol synthase | *Isodon rubescens* | KX831650 |
| IrTPS3 | (+)-copalyl diphosphate synthase | *Isodon rubescens* | KX831651 |
| IrTPS4 | miltiradiene synthase | *Isodon rubescens* | KX831652 |
| IrTPS5 | *ent*-copalyl diphosphate synthase | *Isodon rubescens* | KX831653 |
| IrTPS6 | uncharacterized diterpene synthase | *Isodon rubescens* | KY661362 |
| IrTPS7 | uncharacterized diterpene synthase | *Isodon rubescens* | KY661363 |
| MvCPS1 | peregrinol diphosphate synthase | *Marrubium vulgare* | KJ584450 |
| MvCPS3 | (+)-copalyl diphosphate synthase | *Marrubium vulgare* | KJ584452 |
| MvEKS | *ent*-kaurene | *Marrubium vulgare* | KJ584453 |
| MvELS | 9,13-epoxy-labd-14-ene | *Marrubium vulgare* | KJ584454 |
| *Pp*CPS/KS | *ent*-kaurene/kaurenol synthase | *Physcomitrella patens* | BAF61135 |
| RoCPS1 | (+)-copalyl diphosphate synthase | *Rosmarinus officinalis* | KF805857 |
| *Ro*KSL1 | miltiradiene / manoyl oxide synthase | *Rosmarinus officinalis* | KF805858 |
| *Ro*KSL2 | miltiradiene / manoyl oxide synthase | *Rosmarinus officinalis* | KF805859 |
| SdCPS1 | *ent*-copalyl diphosphate synthase | *Salvia divinorum* | KX424876 |
| SdCPS2 | clerodienyl diphosphate synthase | *Salvia divinorum* | KX424877 |
| SdKSL1 | Multi-product diterpene synthase | *Salvia divinorum* | KY057342 |
| SdKSL2 | uncharacterized diterpene synthase | *Salvia divinorum* | KY057343 |
| SdKSL3 | uncharacterized diterpene synthase | *Salvia divinorum* | KY057344 |
| SfKSL | miltiradiene synthase | *Salvia fruticosa* | AJQ30185 |
| SmCPS1 | (+)-copalyl diphosphate synthase | *Salvia miltiorrhiza* | EU003997 |
| SmCPS2 | (+)-copalyl diphosphate synthase | *Salvia miltiorrhiza* | JN831114 |
| SmCPS3 | inactive enzyme | *Salvia miltiorrhiza* | JN831115 |
| SmCPS4 | *ent*-labda-13-en-8-ol diphosphate synthase | *Salvia miltiorrhiza* | JN831120 |
| SmCPS5 | *ent*-copalyl diphosphate synthase | *Salvia miltiorrhiza* | JN831121 |
| SmKSL1 | miltiradiene synthase | *Salvia miltiorrhiza* | EF635966 |
| SmKSL2 | epi-manoyl oxide synthase | *Salvia miltiorrhiza* | JN831119 |
| SsLPPS | labda-13-en-8-ol diphosphate synthase | *Salvia sclarea* | JQ478434 |
| *Ss*SCS | sclareol synthase | *Salvia sclarea* | JQ478435 |
