# Supplementary material for: Biosynthesis of the oxygenated diterpene nezukol in the medicinal plant Isodon rubescens is catalyzed by a pair of diterpene synthases
Source: PLoS One. 2017 Apr 26;12(4):e0176507. doi: 10.1371/journal.pone.0176507 (PMC5405970; doi:10.1371/journal.pone.0176507)
Supplement: S2 Table — (PDF) [file pone.0176507.s002.pdf]

**Table S2:** Oligonucleotides used in this study.

| Designation | Gene | Sequence (5’-3’) |
|---|---|---|
| pET28b(+) | IrTPS2-For | TATCATATGTCTACTCTCAAACTAATCCCCTTC |
| | IrTPS2-Rev | TATGGATCCCTAATGAGTTGAAAGATGAGGAA |
| pLIFE33 | IrTPS2-USER-For | GGCTTAAUAATGTCTACTCTCAAACTAATCCCC |
| | IrTPS2-USER-Rev | GGTTTAAUCTAATGAGTTGAAAGATGAGGAAGG |
| | IrTPS3-USER-For | GGCTTAAUAATGGCTTCTCTATCAACTATGCAC |
| | IrTPS3-USER-Rev | GGTTTAAUTCACACGACTGGTTCGAAAAG |
| | IrTPS4-USER-For | GGCTTAAUAATGGGGAAAATTAAAGAGAAGTTCAATG |
| | IrTPS4-USER-Rev | GGTTTAAUCTAATCTATGTAACATGTCATCATGACATTTG |
| | IrTPS5-USER-For | GGCTTAAUAATGTCGTCTTCTTCCATTGTC |
| | IrTPS5-USER-Rev | GGTTTAAUCTAATCTATGTTTTCGAACAGTAC |
| qPCR | IrTPS1-For | CGGCGAGTTCTTCTGTTTC |
| | IrTPS1-Rev | CGACGAGGATGTTTTCTCC |
| | IrTPS2-For | CATTAGCCTGTGGGTGGAG |
| | IrTPS2-Rev | GCAGCCGATGGTAACTGG |
| | IrTPS3-For | CTCCAACCTAACCAGCAAAATC |
| | IrTPS3-Rev | TTTCCCAAGGACTAACTTCACC |
| | IrTPS4-For | GATGCAATTCCTCGGTGTG |
| | IrTPS4-Rev | TTTTCCTTGCGTTCCTTCTC |
| | IrTPS5-For | TCGGGGAGTTTGGGTTG |
| | IrTPS5-Rev | CGCTCTCCTTTCTTCTCTGG |
| | IrTPS6-For | TGGTTGAATTATGGGATGTGG |
| | IrTPS6-Rev | ACGCCCTTGTAGCTGGTATG |
| | IrTPS7-For | ACGGTGATGATGAAGTGGTG |
| | IrTPS7-Rev | GGTAGTCCTTGCCTTTGTTGTAG |
| | Actin-For | AGGGTCCATCTTGGCTTCTC |
| | Actin-Rev | CCGACTCATCATATTCTGCTTTC |
