# Supplementary material for: Biosynthesis of the oxygenated diterpene nezukol in the medicinal plant Isodon rubescens is catalyzed by a pair of diterpene synthases
Source: PLoS One. 2017 Apr 26;12(4):e0176507. doi: 10.1371/journal.pone.0176507 (PMC5405970; doi:10.1371/journal.pone.0176507)
Supplement: S1 Fig — (PDF) [file pone.0176507.s003.pdf]

S1 Fig. 8β-hydroxy-sandaracopimar-15-ene or nezukol.
^{13}C and ^{1}H NMR (201 MHz and 800 MHz, $CDCl_3$)

C	δ_C (ppm)	δ_H (ppm), multiplicity	J (Hz)
1	39.4	0.82 (m), 1.71 (dtd)	12.8, 3.4, 1.5
2	18.5	1.40 (m), 1.62 (m)	
3	42.1	1.14 (td), 1.38 (m)	13.8, 13.2, 4.5
4	33.3		
5	56.5	0.85 (m)	
6	17.8	1.51 (m), 1.54 (m)	
7	43.5	1.34 (m), 1.66 (dt)	13.3, 3.2
8	72.7		
9	57.0	0.85 (m)	
10	37.2		
11	17.0	1.49 (m), 1.62 (m)	
12	38.1	1.31 (m), 1.55 (m)	
13	36.5		
14	51.5	1.31 (m), 1.35 (m)	
15	151.7	5.73 (dd)	17.5, 10.7
16	108.5	4.81 (dd), 4.87 (dd)	10.7, 1.3; 17.5, 1.3
17	24.2	1.22 (s)	
18	33.5	0.84 (s)	
19	21.6	0.86 (s)	
20	15.6	0.99 (s)	
