# Supplementary figures and images for: Biosynthesis of the oxygenated diterpene nezukol in the medicinal plant Isodon rubescens is catalyzed by a pair of diterpene synthases
Source: PLoS One. 2017 Apr 26;12(4):e0176507. doi: 10.1371/journal.pone.0176507 (PMC5405970; doi:10.1371/journal.pone.0176507)

S2 Fig. Nezukol 1D selective NOE spectra of Me-20 and Me-17.

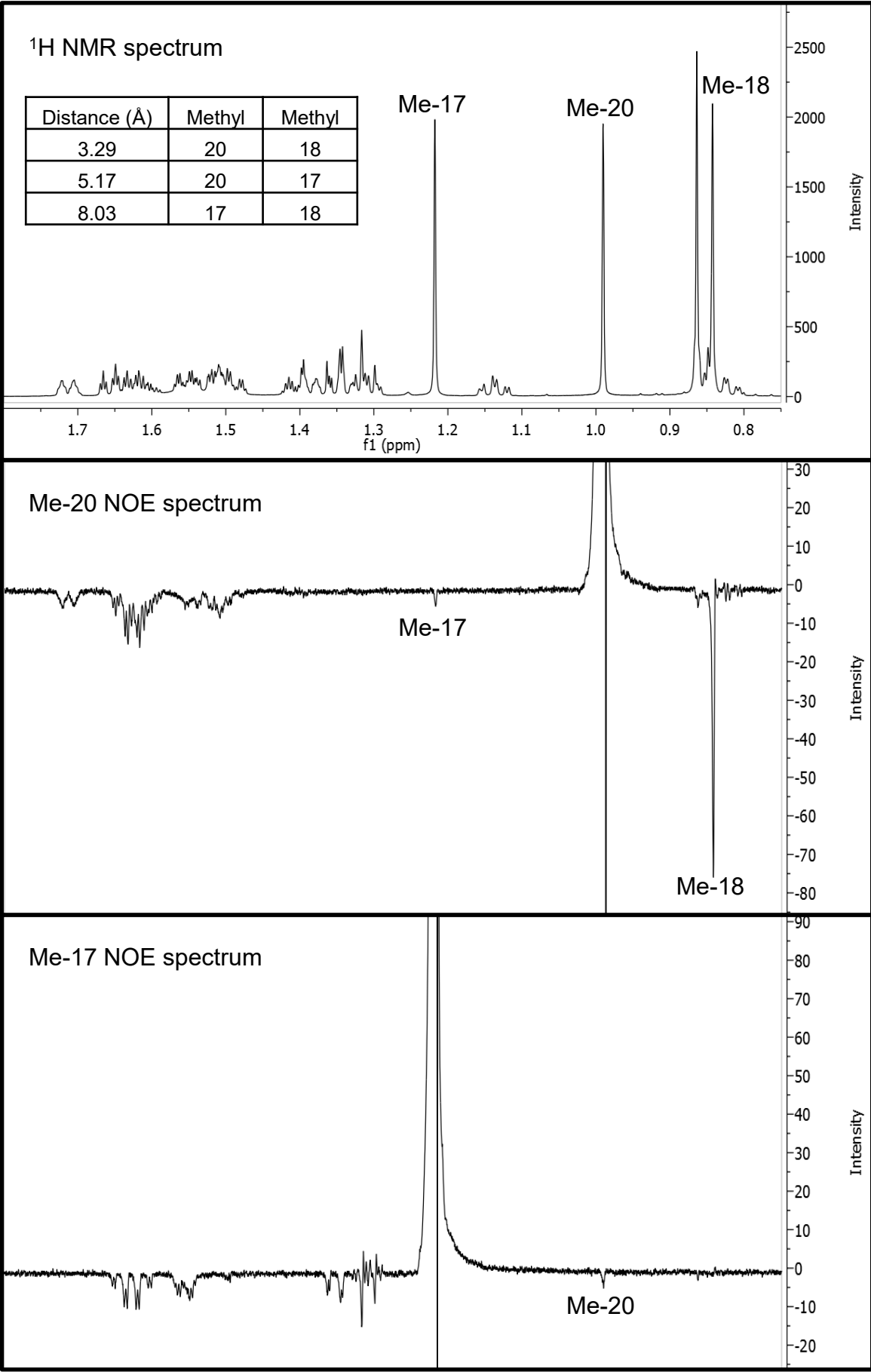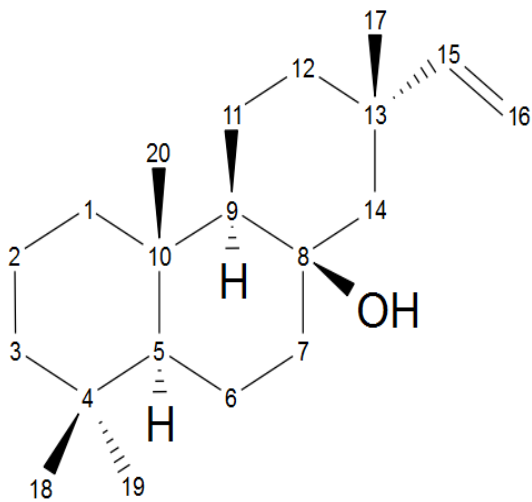

Supplement: S2 Fig — (PDF) [file pone.0176507.s004.pdf]
